# A Critical Realist Approach on Autism: Ontological and Epistemological Implications for Knowledge Production in Autism Research

**DOI:** 10.3389/fpsyg.2021.713423

**Published:** 2021-12-23

**Authors:** Marianthi Kourti

**Affiliations:** Department of Social Work and Social Care, University of Birmingham, Birmingham, United Kingdom

**Keywords:** autism, critical realism (CR), participatory research (PR), autistic theorizing, philosophy of autism

## Abstract

The ontological status of autism has been a subject of considerable debate and philosophical approaches of it have been recent and sparse. On the one hand, from its conception, autism has been historically heavily located in the fields of psychiatry, psychology and neuroscience, which often assume access to an “objective,” neutral and infallible reality that is external to the research process and is based on the autistic person’s biology and behavioural characteristics, which can be scientifically observed and studied. On the other, proponents of the neurodiversity movement argue against medicalised and pathologising approaches to autism and toward approaches that consider social constructions of autism and relations of power. The Critical Realist philosophy can help reconcile the two positions. Critical Realism conceptualises objectivity as a statement about an object, rather than a neutral and infallible reality. Consequently, Critical Realism suggests that access to reality can only occur through fallible theories. It also suggests that effective theorising goes beyond appearances and phenomena and may even contradict them, which can help challenge dominant behaviourist approaches on autism. I then explore how the tenets of Critical Realism can help strengthen autistic-led theories of autism, the arguments they make, as well as how they support the importance of community autism knowledge. Finally, I present how Critical Realism’s approach to knowledge itself as well as the process of knowledge creation can strengthen autistic theorising, autistic participation in autism research and autistic emancipation. In the last part of the article, I explore how the concepts of Critical Realism apply to autistic sociability. I start with the debate between structure and agency, how Critical Realism reconciles this debate and the implications for autistic emancipation and autism research. I then present Critical Realism’s process of critique and explanation, how they connect to human emancipation and how they can lead to impactful change in autism research by requiring clear links from research to practice, enhancing practices with strong theoretical underpinnings and thus aiding the aims of emancipatory autism research.

## Introduction

Autism studies have changed significantly over the decades since autism was first conceived by Kanner, 1943 (Feinstein, 2011). In the first decades since its conception, autism was almost exclusively studied under the field of psychiatry. Autism studies later expanded to also be studied by psychology, neuroscience, and education (Bagatell, 2010). In recent years, and mostly thanks to contributions from autistic activists and writers, the ideas around autism have been also seen through the context of humanities and social sciences, aiming to understand the various ways in which social inequalities shape autistic lives (Pellicano et al., 2018). Autism definitions, therefore, range significantly from a deficit-based approach, such as the definition of the DSM-5 (American Psychiatric Association [APA], 2013), which defines autism as “persistent deficits in social communication and social interaction across multiple contexts, and restricted, repetitive patterns of behaviour, interests, or activities, currently or by history” to its perception as a form of neurodiversity (Singer, 1999). The neurodiversity model ranges from approaches to neurodiversity with a basis in biological and genetic differences (e.g., Singer, 1999; Silberman, 2017) to characterising neurodiversity as a form of social identity and movement aiming at social justice and thus forming the neurodiversity paradigm (e.g., Strand, 2017). These approaches are not mutually exclusive, just differing somewhat from each other in focus and framing. What has, until recently (e.g., Chapman, 2020; Botha, 2021b) been missing, however, is a thorough examination of the underlying philosophical implications of each position, and their implications for the knowledge they create. To understand the current contexts around autism and what they may mean, therefore, it is important to introduce a philosophical approach on autism that is able to incorporate all these different disciplines and bring them together whilst still criticising harmful practices and prioritising the needs, perspectives, and emancipation of autistic people.

Perhaps it is wise to start by examining why we might need one. Firstly, attempts to introduce a philosophy of autism have been sparse, relatively recent and mostly overlooked by the majority of traditional autism researchers. Furthermore, as Richman (Bölte and Richman, 2019) notes, philosophy may not tell us what autism *is*, but it can examine the questions we ask and what these might mean for the answers we gather. A philosophy of autism may be less concerned with findings, and more with the frameworks and means of interrogation used, as well as what they might mean for the conclusions reached. Once these are established, the disciplines that study autism can take over to investigate their empirical aspects. The pursuit of a philosophy of autism is, therefore, a question of framework. As Collier (1994) highlights, it is important to consider the metatheoretical aspects of the work of scientists who are explicitly interested in their discipline which they often “*do not need to make explicit and may not even suspect that they use*.” This pursuit is not concerned with how thorough someone has been in their attempt to do autism research, and indeed the idea that strict adherence to methodology is what produces good research can in itself be harmful (Botha, 2021a). Instead, it is about the underlying meaning behind those attempts and how they might frame research findings.

Complementary to this, Collier (1994) answers the question “Why philosophy?” by noting that the alternative is not a lack of philosophy, but rather a *bad* philosophy. He suggests that someone who may consider themselves to be, or appear to be, an unphilosophical person, still has a philosophy, but this may be unconscious, lack critical awareness and as a result it may be disjointed and inconsistent. The work of a philosophy of autism, therefore, as with philosophy in general, is to highlight what philosophies are implicit in various practices, how they are used by those practicing that science, even when they are not aware they are using them, and to make them explicit so they can be examined and critiqued. The other role of philosophy (Collier, 1994), is tounravel some practices that do what certain *a priori* theories say cannot be done. This is perhaps especially pertinent in the case of autism and traditional conceptualisations of what it means to be autistic. Critical Realism is the name that has been attributed to the works of Bhaskar (1987, 1989, 1975, 2015), a philosopher whose work is mostly concerned with ontology, the study of *being*, and how various disciplines (Cruickshank, 2003), such as sociology, psychology, biology, and feminist theory, approach it. Its name combines the two ontologies that Bhaskar engages with, *transcendental realism*, which refers to Bhaskar’s analysis of the natural sciences, and *critical naturalism*, which refers to the implications of transcendental realism for the human sciences (Archer et al., 2013). Critical Realism aims, to be, therefore, an interdisciplinary meta-theory that explores how science comes to understand the world and how epistemology, the process of knowledge creation, engages with and shapes ontology, our understanding of nature and society (Bhaskar et al., 2017).

Botha (2021b) discusses in further detail how both positivist and interpretivist approaches have been used in psychology generally and autism research more specifically, critically evaluates their shortcomings and explains why Critical Realism is a better alternative. This article will explore how the philosophy of Critical Realism might be applied in autism research, and how it not only supports, but rather requires, autistic participation in autism research. It also considers whether the tensions within autism studies arise from different disciplinary understandings of knowledge and the fact that interdisciplinary research is the exception rather than the rule, even though it is often championed by autistic scholars and activists (e.g., Arnold, 2020).

This article will introduce the philosophy of Critical Realism, give an overview of its main tenets, and will discuss how this philosophy can be applied to autism studies, the framing autism as a concept and knowledge creation in autism studies. Firstly, it will focus on transcendental realism to discuss autistic embodiment, and then on critical naturalism to discuss autistic sociality. I will use examples of autistic-led theory, specifically monotropism and the double empathy problem, its implications and use in various disciplines and their impact in how autism knowledge production. I will also examine how Critical Realism’s concepts can support and substantiate participatory and emancipatory autism research.

While this article is an attempt to encourage discussions around the philosophy of autism, I myself am not a philosopher. I am a social studies researcher who has found it increasingly impossible to continue doing autism research without addressing some of its the ontological and epistemological aspects first. As such, the purpose of this article is to encourage autism researchers, professionals and autistic activists who may also not be well versed in philosophy, to consider the philosophical implications of their positions and reflect on how those impact on their theory, research, practice, and activism. It is also an invitation to those more philosophically inclined than I am to further tease out these concepts further in an accessible, inclusive, and participatory way since, as an autistic individual and activist, these will always be at the core of my approach to autism research.

## Transcendental Realism: Autistic Embodiment

Transcendental realism refers to the part of Bhaskar’s philosophy that is concerned with the study of the natural world, and therefore can be considered as a philosophy for the natural sciences (Bhaskar, 1975). Transcendental realism accepts the existence of an external reality, an intransitive object, that exists regardless of our knowledge of it. However, it also accepts that our knowledge of this object may only approximately describe the intransitive object, therefore our knowledge is subject to fallibility. In this way, transcendental realism aims to reconcile ontological realism, the existence of an interpretation of reality that is fallible and a definitive definition of reality beyond our knowledge claims (Cruickshank, 2004), epistemological relativism, the idea that our approach to knowledge creation as well as our modes of thinking, perspectives, thoughts processes, dispositions interests and values are deeply rooted in our socio-cultural situations and therefore inseparable from them (Lawson, 2003), and judgemental rationality, the process of showing how some claims are more true than others (Wiltshire, 2018). Judgemental rationality is the critical realist process of evaluating a theory in order to establish why it may be better than another theory. By establishing it as more coherent and representative of its subject, it is less contradictory and disjointed, and is preferable because its internal structure is superior and it is more useful and adequate in practice compared to other theories (Scott, 2010). A theory that is realist considers knowledge to consist of objectivity, fallibility, transphenomenality, and counter-phenomenality (Collier, 1994). In the paragraphs that follow, each of these terms will be explained and the relationship between ontological realism, epistemological relativism and judgemental rationality will be further explored. I will then argue why this approach is useful for a philosophically sound theory of autism and how it can support autistic emancipation.

### Objectivity in Transcendental Realism

The term objectivity is a loaded term among critical thinkers and philosophers. In methodologically positivist approaches of science, objectivity, a “neutral” and unbiased observation and recording of a reality that is external, is often treated as a given; one’s ability to research, analyse, and theorise on the world regardless of their own personal position, routinely remains unquestioned (Montuschi, 2016). Furthermore, measuring often means adherence to specific methodological processes that are considered to be the best, or even only, way that knowledge can be obtained (Chamberlain, 2000). On the other hand, interpretivist and constructionist approaches present objectivity as impossible since it declares independence from any knowing or valuing subject and reality itself is presented as inherently dependent on our own perception of it (Kirk, 2012). For transcendental realism, however, this definition of objectivity is itself flawed (Collier, 2003). After all, as Collier puts it *“to be the object of knowledge may be to be the subject of self-revelation*” (p. 134). Objects, therefore, need not be bound to a relative subject; they exist regardless of their relation to any subject, regardless of whether their existence is known at all (Collier, 2003). An object does not suddenly come into existence once its existence is known; that is the ontological realism that transcendental realism defends.

The problem with traditionally positivist, traditionally constructionist/constructivist and traditionally interpretivist definitions of objectivity, therefore, is that they have come to equate subjects to mean “people” and objects to mean “things”; and to conflate ontological concepts with epistemological ones, existence itself with our knowledge of it. On the one hand, positivist science conflates ontology with epistemology by claiming that an objective reality is accessible, measurable, and quantifiable and it is so despite the researcher’s personal subjectivities. However, as Collier (2003) puts it: “*there is no guarantee that something objective will be measurable, and trying to force the unquantifiable into a quantitative straitjacket is subjectivity in the worst sense*” (p. 132). Interpretivist, and constructionist approaches on science, on the other hand, conflate ontology and epistemology by claiming that we cannot know if reality outside our knowledge exists, thus its existence, or lack thereof, is not epistemologically meaningful. What ends up happening, therefore, is that we conflate an object itself with *our* concept of the object, despite the fact that whatever concepts of that object we have are still *our* concepts (Collier, 1994). Critical realism is not the only philosophy to have pointed that out; Hacking (1999), for example, has also come to similar conclusions when examining how social constructs have been used in American sociology and philosophy and provides similar reasons as to why they might not be as useful. Transcendental realism, therefore, defends epistemological relativism as much as it does ontological realism.

The critical realist use of the concept of objectivity, therefore, differs significantly from both of these definitions. For Critical Realism, objectivity refers to what is true independently of any subject judging it to be true (Sayer, 2000). This does not mean that facts are independent of all judgements (in actuality something may be a fact about a judgement), they are merely independent of the judgement of truth, they need not be judged to be true, in order to be true. Furthermore, human judgements themselves are also objective facts (which does not mean they are neutral and infallible, as this is not the definitions of objectivity Critical Realism uses) because they are judgements *about* something. For transcendental realism, there is a causal theory of perception, a causal process that links an object to the perception of it (Collier, 2003). Transcendental realism, therefore, claims that objectivity is a human attitude; scientific endeavour, consequently, ought to aim to bring our perception of its scientific object as close to that object as possible, whereas the object itself exists independently of our perception of it and its existence governs our thinking around it (Kolnai, 1977). That is how transcendental realism links epistemological relativism with judgemental rationality.

This type of objectivity can help us untie a lot of ontological and epistemological knots around the theory of autism. First of all, we do not *need* to know about autism for the phenomena we have come to describe as autism themselves to exist. Autism will be autism independent of who is looking into it or describing it. The states and characteristics *themselves* do not exist because of our descriptions of them. Our understanding of them does depend on those descriptions however, and therefore so does our epistemology of autism. But whatever our epistemology may be, it will always be an epistemology *of* autism, in the sense that it will always be about a set of traits and characteristics that we have currently come to label as autism. This does not mean that we will not adjust, redefine, modify, and even expand what can constitute autistic traits or characteristics. Indeed, it is not even dependent on them being called autism at all. It may be decided in the future that the term “autism” is not a helpful term to describe what we currently use it for, just as it was decided that the term “Asperger’s Syndrome” was not a useful description in the publication of the DSM-5 (Happé, 2011). It may even be decided that the category of autism is too restrictive or too broad; any and all definitions and descriptions will however, still be definitions and descriptions of an intransitive realm that is, and will always remain, independent of its transitive epistemology.

Furthermore, by using the concept of objectivity as it is described in transcendental realism, one can make the argument that a judgement of the common theories of autism is a fact about those theories, because to say that a theory, an epistemological approach to autism, may be inaccurate, or even harmful, is a characteristic attributed to that theory and it is its characteristic regardless of who its attributor is. To say, for instance, that the weak theory of mind theory of autism dehumanises autistic people as Yergeau (2018) claims, would not merely be about the positionality of the author/subject according to a critical realist approach; rather, it would be about the epistemology of the object, the process of the knowledge creation itself, which occurred prior to, and independently of, any subject judging it. This is important because, particularly in the context of autism and the processing and communication difficulties that often co-occur, it is important to acknowledge that that a theory or practice can be harmful to someone even if that person themselves cannot describe, explain, or even understand why this is the case. This does not, however, mean that it is irrelevant to examine why the theory is critiqued by autistic people *specifically*, and why for decades it was (and still largely is) not deemed as harmful by neurotypical researchers. The concept of judgemental rationality is an important one in defending autistic knowledge creation. It has been argued that in order to understand a skill or theory cognitive comprehension is not enough, but bodily and lived understanding is important as well (Isaksen, 2016). It could be argued, therefore, that these critiques come from autistic people because autistic people have access to deeper domains of autism knowledge, as we shall see below.

### Fallibility as a Consequence of Objectivity in Transcendental Realism

To say that a judgement about something is a fact about that something may make a lot of thinkers initially very uncomfortable. This may likely be because, both in lay knowledge and in naïve positivist approaches of science, facts are often considered to be both neutral and infallible. This derives from the belief that facts and values should be kept separately as it is not possible to derive a value from a fact (Gorski, 2013). It is precisely because of these misguided perceptions that interpretivist, social constructionist, and social constructivist approaches of science have aimed to prove that nobody can claim neutrality and infallibility. In the process, of doing so, however, they have created an approach that, if taken to its logical completion, suggests that an object’s existence is subjective to our knowledge of it (Kemp, 2005), even though, admittedly, most thinkers who follow these approaches do not take this extreme stance. This is not the objectivity that transcendental realism defends; it does not aim to prove that theories can be neutral or infallible. In fact, the claim that it makes is that *precisely because* knowledge is objective, it is knowledge *about* an object, it is by default *always* fallible (Cruickshank, 2002). It is the very fact that the object it describes is independent of the theory that describes it, that makes the theory’s accuracy and effectiveness able to be evaluated. This is why fallibility is another core tenet of the critical realist philosophy that goes hand-in-hand with objectivity. Transcendental realism recognises that because theories make claims about what the world is like *independently* of those theories, all theories are *essentially* fallible and, consequently, open to transformation. One example that Collier (1994, 2003) gives to support this position is the description of an event where one human died because of the deliberate actions of another human. It is accurate to say that the first person died, however, it is also accurate to say that this person was killed and, in so far as the second human intended for the first human to die, it is also accurate to say that the first human was murdered. To say that the first person was murdered, however, is both more objective, because it presents more facts about the incident (the loss of life; the fact that the loss of life was done by another; and the fact that the other person intended for the loss of life to happen) *and* less neutral because it paints what happened in a light that is clearly unfavourable toward the second human, the perpetrator of the act.

Fallibility is a really important concept for the philosophy of autism and for critically evaluating autism theories, whether they be biological, medical, psychological, or social ones. The concept of fallibility, the statement that our claims about reality are fallible and biassed specifically *because* they are objective (about an object), is what can lead to accountability and the evaluation of a theory of autism against the existence of autism itself. If the existence of autism was exclusively dependent on our theories of it and autism did not have its own ontology, then there would be no reason to assume that medicalised approaches on autism, for example, are more fallible than the concept of neurodiversity and it can simply be a matter of preference which of the two approaches a researcher will follow. Neurodiversity, therefore, loses its polemic bite; it becomes a concept that should be adhered to because autistic people say so, without explaining why neurodiversity should be adhered to *because* autistic people say so; what is it that autistic people can know better about autism that requires for them to be put at the centre of discussion for autism knowledge to be credible. Transcendental realism, therefore, aims to strengthen the neurodiversity movement and its inherently material and discursive dimensions by putting a focus on fallibility and by making the argument that theories made and/or endorsed by autistic communities, are more objective because they represent autism more accurately than neurotypical theories do.

### Transphenomenality in Transcendental Realism; A Liberation From Appearances

Transphenomenality refers to the claim that knowledge consists of more than appearances. Knowledge does not consist of simply how things look, but also of underlying structures that last longer than appearances and make those appearances possible (Steinmetz, 1998). The knowledge we have is not just a knowledge of phenomena, but that of underlying factors and conditions that make those phenomena possible. It is that deep knowledge, therefore, that has explanatory power over the phenomena, over the surface-level realism of observation (Roberts, 2001).

Transphenomenality is an important tenet to consider when looking into the epistemology of autism and the agents of knowledge creation that surround it. Given that much of mainstream understanding of autism is a behaviourist one, it is worth examining what kind of understanding of autism it is; is it just a knowledge of phenomena, or does it (or can it) include the knowledge of the underlying causes and conditions as well? Do the mainstream theories of autism entail the deep knowledge that has explanatory power over behavioural observations, or are they surface-level observations that claim to explain more than they actually can?

It can be argued that many of the “traditional” theories of autism do not stand up to philosophic scrutiny because while they may certainly make claims about what the underlying structures and mechanisms of autistic behaviours are [a weak theory of mind (Baron-Cohen et al., 1985); difficulties in central coherence (Happé and Frith, 2006); extreme male brain (Baron-Cohen, 2002)], they only deduce those mechanisms from behavioural (therefore phenomenal) observations. A neurotypical researcher or clinician, therefore, can only rely on their own assessments of autistic behaviours to draw conclusions about what autism *is*, thus making their knowledge of autism a knowledge about *phenomena* of autism, not a knowledge of the underlying factors and mechanisms that cause the phenomena. It is, therefore, by default a shallow, surface-level knowledge of autism.

It should be noted here that this is the case despite methodological rigour on behalf of the researcher. “Shallow” does not mean poorly researched, at least not in so far as methodological rigour is concerned; it simply means that no matter how thorough the research is, it can only ever be research about what autism *looks like* and not what autism *is*, despite any claim to the opposite or attempt to conflate the two. If there is a concession among the scientific community, which at large still seems to be the case, that autism needs only be examined on the basis of lack of social skills, poor theory of mind, lack of central coherence, and an extreme male brain, then no matter how thoroughly a researcher looks into those phenomena, it is only ever those phenomena that are being looked into, not their causes. The phenomena are then explained based on perceptions that are created through observation, and those explanations are mistakenly referred to as causes. This is what a Critical Realist philosophy of autism can help us disentangle, and further address, since, as it was stated earlier, it is not interested in examining how the cognitive results of science are achieved, but rather what concepts are implicit in them, regardless of the philosophical stance of the researcher (or lack thereof) and how these concepts can be made explicit so they can be evaluated and critiqued.

### Counter-Phenomenality in Transcendental Realism; When Circumstances Contrast Appearances

Counter-phenomenality refers to the idea that knowledge about the deep structures of a theory may not just simply explain appearances, but also contradict them (Collier, 1994). This idea is not new to Critical Realist philosophy; however, it is a fundamental tenet of it. According to Critical Realist theorising, it is the counter-phenomenality of knowledge that allows us to go beyond appearances, rather than stay bound to them. Counter-phenomenality is important for our liberation from appearances, because, as Marx has stated, appearance being something different from essence (Reichelt, 2005) is an essential presupposition of science, or else science itself would be redundant.

Counter-phenomenality is important to consider when engaging in autism theory, because all the mainstream theories of autism do not consider it. The main rationale behind them is that if autistic people *appear* to lack theory of mind, central coherence, have an extreme male brain etc, then they must *really* lack or have all these qualities as demonstrated in the assessment/questionnaire/parental interview etc. and interpreted by the neurotypical researcher/professional. Because autism knowledge is behaviourist, surface-level and phenomenal, it does not account for what the embodied experience of being autistic might actually *be* like, and only relies on appearances to provide explanatory theories of autism. But a theory that lacks deep realism, a theory that does not attempt to be counter-phenomenal or to consider counter-phenomenality to be possible, can only ever be a theory about appearances, and thus shallow realism.

The argument that I am making here is that the predominant theories of autism do not stand up to philosophic scrutiny, not because they are neurotypical, but because they consist of behaviourist observations that only represent a shallow reality, phenomena, rather than the deeper realities of events and mechanisms, to which they do not, and cannot, have access to. The reason, however, that they do not and cannot have access to them is *because* they are neurotypical, and therefore do not know what the experience of living an autistic life in an autistic body is like. Those experiences are only ever accessible to autistic people, whether they can communicate them or not. Although it is important to account for the heterogeneity among autistic people and the fact that there may be many non-autistic people who share some of their experiences whether embodied (an impairment with similar presentation for example) or social (the experience of being marginalised), this does not negate the fact that autistic people are forming connections based on the recognition of such similarities in each other. Below I will attempt to explain how the philosophy of Critical Realism supports this assertion and what this might mean for the epistemology of autism.

### Transcendental Arguments and Community Autism Knowledge

Transcendental realism introduces transcendental arguments, arguments that attempt to epistemologically transcend the “shallow,” surface-level reality of phenomena and instead explain events and mechanisms that cause the phenomena, or experiences, to happen. For Critical Realism, reality consists of three domains: the domain of the empirical, which consists of experiences, the domain of the actual, which consists of experiences and events, and the domain of the real, which consists of mechanisms, events, and experiences (Bhaskar, 1975). According to Bhaskar (1975), the domain of the actual is greater or equal to that of the empirical and the domain of the real is greater or equal to that of the actual. Mechanisms, therefore, have greater explanatory power than events, which in turn have greater explanatory power than experiences.

Transcendental arguments can have significant impact on how we view a critical realist philosophy of autism. As it was stated before, the predominant understanding of autism is a behaviourist, neurotypical interpretation of autistic behaviours and relies, therefore, on the shallowest, most surface-level domain of reality to understand autism; that of experience. The autistic understanding of embodied autism has access to the domain of the empirical and the actual, to both experiences and events. The reason for that is simple, the events themselves manifest within our own bodies. When an autistic person has a meltdown, for example, a non-autistic person can only understand it by witnessing it; the event itself, the meltdown, happens within their autistic body and therefore only the person themselves has access to any information about it (how it feels, how it progresses, what might help etc.). To claim that any non-autistic person has access to the domain of the actual when it comes to embodied autism would be to claim that a person who is not the person themselves can have access inside their body, which obviously is impossible for any human. Of course, autistic people do not understand each other because of some sort of “magical” access to each other’s bodies. We can simply interpret each other’s experiences, the empirical, with information we draw from both the empirical and the actual; drawing from both information on our own embodiment, which is more likely to have similarities to each other’s than a neurotypical person’s embodiment has to our own (events) *and* from our interactions with each other (experiences). This is also why tokenistic practices are counterproductive; the phrase “when you’ve met one autistic person, you’ve met one autistic person” is a cliché for a reason; no single autistic person could ever provide a credible theory of autism in isolation. It is in a community of autistics, therefore, that autistic knowledge is created, and it is this community knowledge that is a more philosophically credible autism knowledge.

Mechanisms, according to Critical Realism refer to the “causes” of phenomena, what causes phenomena to occur. Each scientific discipline then approaches and explains those mechanisms using a different lens. In genetics, therefore, the causes of autism might be located in the DNA, in neuroscience they would be located in different functions of the brain and the nervous system, in psychology in behaviours, in sociology in societal structures, how they classify various humans and how social practices affect autistic people, in humanities how autism may be presented through various art forms etc. For Critical Realism, reality is stratified and each scientific discipline studies a different stratum of it (Joseph, 1998). Furthermore, many events are not caused by a single mechanism, but by a variety of mechanisms taking place at the same time; we are all, for example bound by the laws of physics and the laws of physics can explain, in some form, most phenomena, but that does not mean that they can explain them fully, as many phenomena, such as functions in the human body, for example, will be also bound to the laws of chemistry and biology (Bygstad et al., 2016). Furthermore, there are no “original” causes; causes have causes (Fairclough et al., 2002). This is important in the case of autism, because significant effort, and funding, has been put into identifying the causal mechanisms that may be linked to (I would argue some) autistic presentations; this has currently not been identified, and many believe that it is unlikely to ever be, but even if it was, it would only be the cause of autism in so far as genetics were concerned, while other scientific disciplines would have other explanations about the causes of autism that would approach the phenomena of autism from the point of view of their field.

Even within genetics, however, identifying a specific genetic sequence is only part of the story; we already know, for example, that Down Syndrome is caused by trisomy 21 on a genetic level (Hultén et al., 2008). But we do not fully know what causes trisomy 21; there are some suspicions, age of the gestational parent being one example, but nothing that fully explains when and how trisomy 21 manifests. Even if these causes were identified, we would have to find the causes of *those* causes and we might have to look further than genetics to do so; many of the reasons that people get pregnant later in life, for example, will be better studied through the social sciences. Further, it will be pertinent to examine the motives and practices behind discovering the genetic mechanisms, which will also be done through disciplines like psychology and sociology. Putting the end of the search for causation at the genetic level, therefore, is somewhat arbitrary and certainly only has partial explanatory power.

The three domains of Bhaskar’s deep realism are important to consider in the context of autism studies, because we have to establish a) whether research is studying experiences or events and b) what kind of mechanisms might have better explanatory power over what kinds of events and experiences. In so far as mechanisms refer to the genetics of autism for example, the causes of autism are currently unknown; the question, therefore, that can be posed is what research that is looking into the genetics of autism is actually researching. An argument can be made that if, as Bhaskar (1975) states, the domain of the real is greater or equal to that of the actual which is greater or equal to that of the empirical, the domain of the actual cannot be skipped if mechanisms are to be established; it is impossible, therefore, to “jump” from the experiences to the mechanisms without understanding the events, and therefore it is impossible to discover the genetics of autism without taking autistic perspectives into account.

I am not discussing the genetic mechanisms of autism here to encourage research on the causes of autism; this is justifiably not an autistic priority and there are some understandable anxieties about how this knowledge will be used in an ableist society (Chapman and Veit, 2020). Furthermore, as already established, the mechanisms that may have explanatory power over the phenomena of autism cannot solely be found in any one discipline; understanding social and economic structures will also contribute in our understanding of autism as it is today by investigating, for example, how capitalist structures focussed on productivity and output may approach individuals whose embodied state of being does not conform to their demands and how this may shape research interests of that embodied state of being (Broderick and Roscigno, 2021). What is argued here instead is that the mechanisms of autism would have to be investigated from various disciplines if we are to have a coherent picture, and it would have to include autistic input if it is to be research that explains events as they are embodied as well as experiences as they are observed, which is crucial for impactful theorising. These are all important considerations given the highly disproportionate funding that some types of autism research receive over others and the significant lack of autistic input in autism research overall, which are both based in perceptions that are ontologically inconsistent, epistemologically problematic, and ethically hard to defend.

What transcendental arguments can help establish, therefore, is that autistic people have access to deeper domains of knowledge compared to non-autistic people. This is independent of an individual’s ability to communicate, contextualise or even understand that knowledge. The knowledge is there regardless of whether it is consciously understood. For example, I knew I was feeling anxiety years before I knew that what I was feeling was called anxiety and I knew that I had this feeling despite not knowing what it was called; however, learning that I am autistic and getting in touch with other autistic people helped me not only put a name to the feeling (event) of anxiety, but also to understand some of the contributing factors to it, to have a partial understanding of its mechanisms (an understanding that, for me, is only ever going to be somewhat partial). What helps conscious understanding of that knowledge, therefore, is interaction with other autistic people whose bodies manifest similar events (meltdowns, sensory sensitivities, monotropic focus etc.). Consequently, autistic communities are fundamental both for autism knowledge that is more credible to that of any individual autistic person’s, and for autism knowledge that is deeper than what neurotypical people, individually or collectively, can produce. Furthermore, community autism knowledge is knowledge that can in great lengths explain any one individual’s autistic experience, even if the person themselves may not be in a place to do so at a particular moment in time (Kapp, 2020). For anybody, therefore, who struggles to understand an autistic person, or for an autistic person who struggles to understand and/or express themselves, community autism knowledge can greatly (although not completely!) fill some of those gaps in knowledge.

Community autism knowledge is also important because it can help respond to the “you are not like my child” argument. Neurotypical parents (and professionals) who have (or work with) autistic children (or people) with learning difficulties often claim that autistic knowledge creation, such as the concept of neurodiversity, does not take into consideration the kind of autism that their child “has”^1^ (Hillary, 2019, 2020). A transcendental argument in response to that claim would be that autistic community knowledge applies to their child partially, but not completely, as much as it partially, but not completely, applies to any one autistic person. It is also a surface-level understanding of autism that only views autism as a set of observable behaviours, and therefore lacks the transphenomenality and counter-phenomenality that a critical realist approach can provide. Autistic people can, to an extent, understand the events that happen within the body of an autistic person with learning difficulties because they can draw information from both their own autistic bodies (the actual) and interactions with other autistic people (the empirical) and then apply this type of community autism knowledge to the specific autistic presentation of that person. They provide, therefore, an understanding of that person’s behaviours that, although inevitably incomplete, will nonetheless be more credible from that of a non-autistic person.

### Transcendental Arguments in Autistic Theorising

Autistic theories such as the Double Empathy Problem (Milton, 2012) and the Monotropism theory (Murray et al., 2005) can be two examples of transcendental arguments that, from a Critical Realist standpoint, provide a theory of autism that is deeper than the neurotypical counterparts they are responding to. In the following paragraphs, I will explain why this is the case and what practical implications it might entail.

The Double Empathy Problem (Milton, 2012) is a theory that was developed as a response to the prevalent neurotypical explanation of autism that being autistic entails a lack of theory of mind and a difficulty, or inability, to empathise. Milton (2012) critiques the common tendency of autism research most commonly found in the fields of neuroscience and psychology to present a set of behaviours as the norm and aim to suggest “treatments” that aim to bring behaviour that is deemed to deviate from that norm as close to it as possible. It suggests that these approaches ignore core components of communication, such as relationality and interaction, when in actuality communication is a two-way street, meaning that autistic people may not communicate effectively with neurotypical people, but neurotypical people communicate just as ineffectively with autistic people, thus presenting a double empathy problem.

The theory of monotropism (Murray et al., 2005) discusses the distribution of attention in autistic people. It argues that every person has limited attention at their disposal, however, how different people distribute that attention may differ according to neurotype. Namely, non-autistic people tend to have polytropic modes of attention, meaning that they distribute a little bit of attention many different places. Autistic people, on the other hand, tend to have monotropic modes of attention, meaning that they tend to give most of their attention in few sources at a time, or even in one. They argue that this is a core characteristic of autistic processing, and that it may account for what is commonly perceived by non-autistic researchers as weak central coherence, which they use to mean difficulties in putting information together and in processing information in context. They also argue that this monotropic attention focus may account for some of the sensory integration difficulties autistic people experience, thus making it a core characteristic of the condition.

Both these theories are good examples of transcendental arguments because even though they were not conceptualised as such by the original authors, they fulfil all the tenets of a transcendental argument: they are objective, because they are judgements of the theory of autism that compare autism theory to its object, autistic embodiment, not to a feature of their subject, the perceptions of neurotypical researchers, whose only relation to the object is the empirical experience of its effects; they call for fallibility, because they are attempting to transform existing structures by providing a less fallible framework on which autism can be considered; they are transphenomenal, because they do not rely on shallow, surface-level behaviourist and cognitive criteria to describe autism but rather go beyond those to describe the events that affect those criteria and how these manifest in the autistic body; finally, they are counter-phenomenal, because they argue that despite the fact that autism may be perceived as lack of theory of mind and weak central coherence to a neurotypical observer, it can actually be better understood as a monotropic use of attention and a double empathy problem in interactions between people of different neurotypes.

Some recent studies have, either explicitly or implicitly, further substantiated these theories with empirical research. By looking at them we can understand why autistic theories on the mechanisms of autism have greater explanatory power than their neurotypical predecessors. Heasman and Gillespie (2018), conducted research that investigated how autistic people and their non-autistic family perceived their misunderstandings. They asked their participants to rate various aspects of their relationship in terms of themselves, the other person, and the predicted rating of the other person. By doing so, they identified that autistic people were able to accurately predict what their family members may think about them despite the fact they disagreed with them, whereas family members tended to overestimate how much their autistic relatives will be stuck in their own perspective.

In a recent paper that was published by Crompton et al. (2020) it was noted that rapport between individuals was dependent on neurotype matching rather than being autistic or not. Specifically, two separate studies investigated rapport in couples that were either autistic, non-autistic or mixed while performing specific tasks or having informal conversations. The rapport was self-rated and rated by observers of various neurotypes and in both cases it was reported that neurotype matching provides higher evidence of rapport, both self-reported and observed. The researchers explicitly suggest that the two studies support the Double Empathy Problem theory.

Goldknopf (2013) investigates aspects of the monotropism theory that have to do with resource allocation making, although not explicitly, many links between the current literature available on autism and atypical attention resource allocation. Specifically, atypical resource allocation is linked to differences in shifting and breadth of attention, movement, executive function and various aspects of language and communication, social cognition and interaction, therefore making resource allocation (and thus monotropism) a central characteristic of autistic dispositions. Ashinoff and Abu-Akel (2021) also examine hyperfocus, which has many commonalities with the theory of monotropism, and highlight the benefits on investigating this state further. They also note, however, that are many challenges in doing so, including the different disciplinary approaches to the concept as well as practical difficulties in clinical research that would engage with it. Wood (2021) examines how using monotropic interests in school can help develop a variety of skills in autistic children. Similarly, Leatherland (2018) explores how the monotropism theory is key in understanding the experiences of autistic secondary school pupils. Both of these papers give monotropism a central place in their investigative efforts and report that engaging with the theory gave their data great explanatory potential. It is important, therefore, for more autism researchers to consider putting autistic-led theories in the forefront of their research agenda to further examine their explanatory abilities.

The debate between Milton and Timimi (2016) on whether autism has an essential nature can be seen as another example of the importance of transcendental arguments for impactful theorising. In it, Timimi claims that autism does not have an essential nature, also argued elsewhere (Timimi, 2011; Timimi and McCabe, 2016), frames any autistic identification and culture under a medicalised framework, and states that the idea of neurodiversity is useful only in terms of eradicating the stigma around autism and does not have meaningful explanatory properties. Milton’s responses frame autism as a social construct and a spectrum of dispositional diversity and embodied experience (also argued in Milton, 2014, 2017; and elsewhere), by highlighting autistic contributions and the links between the concept of neurodiversity, autistic culture, and their importance in empowering autistic people to understand themselves away from medicalised discourses.

By considering the characteristics of transcendental arguments, one can clearly identify certain ontological pitfalls in Timimi’s approach on autism. He claims that there are no “essential and knowable biological differences,” thus equating “essential and biological” with “knowable,” falling into constructionist traps that were challenged earlier in the article. Further, he states that “you can’t un-diagnose someone with heart failure, but you can un-diagnose someone of autism,” which is debatable; one may be “un-diagnosed” with heart failure either by medical error or, more nefariously, intentionally by a doctor fearing a ruined reputation that may follow links to ineffective treatment. The latter would of course constitute malpractice, and if discovered it may be punished, but that is independent of the action itself. Similarly, one may be un-diagnosed as autistic simply because they were misdiagnosed in the first place, by an error in a clinician’s judgement, or because a clinician does not think that knowing they are autistic will be of value to them. The latter two could be equally harmful as the first example, since the person would be experiencing the many consequences of being autistic in a neurotypical world, regardless of diagnosis, as many later-identified autistic people will attest. Medicalised approaches do stigmatise autistic people (Grinker, 2015), but the concept of neurodiversity can, and does, help many understand themselves better. Not *all* autistic people will be as invested in understanding autism as some of us are, but this does not devalue the neurodiversity paradigm as an academic approach or a tool for autistic emancipation.

Framing autism as a psychiatric invention to pathologise a set of behaviours neglects that those behaviours pre-existed their pathologisation and therefore can exist outside it. Furthermore, because this framing rejects objectivity, it also attempts to escape fallibility and accountability; interestingly, Timimi does not perceive his approach to autism and his role as a diagnostician as contradictory to one another. Additionally, it is not transphenomenal, because it uses appearances to make ontological and epistemological assumptions about autism by attributing its argued essential inexistence simply to its behaviourist diagnosis. Finally, it is not counter-phenomenal, because by not going past appearances, it also does not consider factors that contradict them.

Milton’s responses align very closely with the arguments presented earlier, which meet the criteria of transcendental arguments as I presented above; as far as this discussion is concerned, therefore, the only element that, in my view, weakens his argument is its lack of an explicitly Critical Realist stance. By presenting autism as a social construct, despite acknowledging the existence of embodied diversity, and not untangling the ontological and epistemological implications of this position clearly, he falls into the trap of engaging with red herring questions such as “how do you know that autism exists?” and cannot meaningfully argue why his position is stronger on any other front apart from ethics, which is heavily critiqued by Timimi throughout. Transcendental arguments, therefore, can strengthen his position by asserting that autism indeed has an essential nature even if it cannot be epistemologically accessed, measured, and analysed.

### Critical Realism, Interdisciplinarity, and Stratification and Emergence

In the previous section I argued that for theories of autism that are reflective of the deeper realism of autism and thus have greater explanatory power, it is important to include autistic input in our theorising to produce strong, transcendental arguments. In this section, I will discuss how Critical Realism as a philosophy can help set the foundation for effective interdisciplinary autism research. I will present how some of the current approaches on autism conflate autistic input with disciplinary approaches to knowledge. Finally, I will explain why using both interdisciplinarity and substantial autistic participation in autism research are important for an understanding of autism that is as complete as possible.

To understand what interdisciplinarity is, we must first establish how different disciplines are divided, why these divisions exist in the first place, what kind of explanatory power over phenomena the mechanisms that each discipline studies have, and how, by interacting with each other, they can capture a fuller picture (Wiltshire, 2018). Critical Realism uses the terms deep and shallow realism because it views reality as stratified. For Critical Realism, reality consists of a number of strata, some more fundamental than others. These strata are not reducible to one another, and a stratum being more fundamental does not mean that it can explain everything found in subsequent strata (Bhaskar, 1998). For example, physics, which is considered to be the most fundamental stratum from which all subsequent strata develop, is not able to fully explain the behaviours of all plants and animals, even though they are all bound by the laws that physics is concerned with. The most helpful way to perceive the stratification of nature, therefore, is a stratification of mechanisms. At the level of the Actual, however, relations between strata overlap, interact, and affect each other in a multiplicity of ways (Collier, 1994).

To return to the earlier example of an autistic meltdown, for example, one may be able to understand and record its physiological elements both as factors that constitute it and as elements that can partially explain it. However, autistic meltdowns may also have social reasons, psychological reasons, sensory reasons, and be the result of other intersecting experiences, and the overlap of all these factors is likely to be unique in each case. Bhaskar examines the relations between mechanisms that reside in different strata in terms of rootedness and emergence. Higher-level mechanisms are rooted in, and emergent from, more basic ones; rootedness, however, does not mean reducibility, because more basic strata cannot explain higher-level mechanisms. While there may be an argument to be made that current social structures around autism emerged originally from the embodied differences of autistic people that neurotypical people tried to regulate, the social structures themselves cannot be fully explained by these embodied differences, as mechanisms rooted in social structures, systems, beliefs and values play a huge part in how these embodied characteristics were perceived and managed.

Another element of higher-level strata is that they cannot be understood as closed systems. Closed systems are what makes experimentation possible, and they are more prevalent in lower strata, such as physics and chemistry. This may be less and less possible when it comes to higher and higher strata, which is why studying mechanisms in those strata means that are used for mechanisms that reside in lower strata may be impossible (Wikgren, 2005). On this basis, Bhaskar (1975) concludes that a person’s neurophysiology is not a closed system, as it is constantly affected by our interaction with others. This could explain why randomised control trials, a method regularly used in the field of psychiatry and psychology to study autism interventions (Simonoff, 2018) are often criticised. Since autism is identified by behavioural criteria, it is dependent on interactions to be observed. It would be impossible, therefore, for it to be studied as a closed system, because the social nature of that interaction is the very thing that is “intervened” on, and interaction cannot be conceptualised as a closed system as it is always susceptible to external factors that cannot be isolated without the phenomenon itself either changing substantially or seizing to exist overall.

The philosophy of Critical Realism can help us address these epistemological inconsistencies and, consequently, to support interdisciplinary research that can provide deep explanatory theories of autism. To do so, we would need a philosophy that can be applied to all disciplines involved, and, as I hope I have shown here, Critical Realism can be just that. The debates between positivist approaches and interpretivist/social constructivist approaches on autism are often presented as differences between disciplines (e.g., Milton, 2012), making communication between disciplines that much more difficult, and further creating the illusion that the knowledge of one discipline is irrelevant to the knowledge of the other. Knowledge and epistemological ways of acquiring or constructing it often does not crossover from one field to another and scientists hold strong and passionate opinions around the impact and validity of their stance, making this gap even harder to breach (Baringer, 2001). Yet studying an all-encompassing set of phenomena such as autism can surely not be done in the constricts of any one discipline alone. Critical Realism can, therefore, serve as a useful meta-theory that can help communication between the various disciplines, the application of the knowledge of one to the other, and help scientists who wish to examine their own practice philosophically communicate with each other more easily (Bhaskar and Danermark, 2006).

This could be the trap that many of the autistic approaches to autism research have fallen into. In their effort to distance themselves from pathologizing approaches to autism, they have conflated autistic input with certain disciplinary approaches. This is mostly because to this day most autism research does not have either; it is both neurotypically produced *and* rooted in only a small subsection of disciplines (namely psychology, psychiatry, neuroscience, and genetics) which applies mainly positivist approaches to its research, and therefore responses to it can easily conflate lack of autistic input with disciplinary approaches that need not be, and often are not, autistically created. Even though the predominant theories of autism that came out of the fields of psychiatry, psychology and neuroscience are not autistic-led, the theories that social scientists use to criticise the pathologisation of autism, and to portray it from the perspective of their discipline, will not be autistic-led *either*; they were likely written by neurotypical researchers to discuss aspects of life that, according to them, apply to neurotypical and neurodivergent people alike, since that distinction was not considered at all. This does not mean they are not useful; it merely means that they are as neurotypically produced as the theories in other disciplines. There is, however, a reason that many autistic scholars are attracted to them; they can be very helpful in the process of explaining aspects of the autistic experience that are not considered at all from research produced in the medical/natural disciplines, thus providing much-needed nuance, and pointing out that the epistemological conceptions of autism through these disciplines are not the be-all-end-all of what autism is, and in fact because they present it as such autism knowledge can easily be grossly misrepresented, as already explained in the prior sections. It is not, therefore, *just* autistic input they need, but interdisciplinary approaches as well.

Although autistic approaches to autism that lie outside the fields of social sciences and humanities may be rare, that does not mean that they do not exist. One such example is the recent paper by Buckle et al. (2020) on inertia, a concept used by autistic communities to describe the difficulty that autistic people may experience in starting tasks, stopping tasks, and switching from task to task, which has not been explored at all by neurotypically led research. In it, Buckle, an autistic neuroscientist, investigates the participants’ experiences of inertia, an interest she developed based on her own experiences of it. In this paper, inertia is presented at least partly as an impairment, rooted in the body, and as something that a purely social constructionist approach on autism may not be able to fully capture or explain. This paper can be viewed as an example that autistic perspectives can be found in any discipline and that to study and understand the embodied experience of autism does not necessarily mean to stigmatise it, regardless of the fact that this is what most neurotypically produced research on autism has historically done. To have a better understanding of autism, therefore, we need both autistic input, which will help us understand the embodied phenomena of autism *and* interdisciplinary approaches, which will help us apply a variety of mechanisms traditionally studied by different disciplines to it in a way that does not stigmatise autistic people but produces a fuller picture of autism instead.

## Critical Naturalism: Autistic Sociability

### Autism, Agency, and Social Structures

So far in this paper, I have used the concepts of transcendental realism to explore how they can be applied to both autism as an embodiment and to the conceptualisation and study of it as an embodied state. In the following part, I will discuss how the second part of Bhaskar’s theory, Critical Naturalism, can be used to understand autistic experiences in a sociological context, what it may mean for how we conceptualise autism and its implications for participatory research.

In his conceptualisation of social beings and social knowledge, Bhaskar (2015) engages with the debate of structure versus agency as it is conceptualised by humanist and structuralist approaches. Humanist approaches examine society purely through the lens of agency and, consequently, as a collection of actions enacted by its agents. Structuralism, on the other hand, sees structure as everything, and considers individuals to be bound by those structures that act in ways that make all agency bound to its relevant structures (Archer, 2003). Bhaskar, following on Marx’s footsteps, reconciles those two positions; he suggests that what is needed, instead, is a “this and” theory, one that considers both agency and structure as aspects that shape society. On the one hand, agency can be seen in human actions committed by either individuals or organisations, such as corporations or governments. On the other hand, the meaning of actions, their functions and limits are decided by societal structures; an agent can only act in so far as the limits of the structure will allow. Bhaskar (2015) suggests, therefore, that to understand those structures we must focus on the *relations* between individuals, between individuals and structures, and the relations between these relations. These relations may be ontologically independent, in that they exist before any one person enters them; however, they are also transformed by the actions of the agents that occupy them. In this way, societies make people and people make societies (Archer, 2000).

This conceptualisation of agency and structure can help us conceive the tensions that may arise both between autistic and non-autistic people, and between neurotypical conceptualisations of autism and their autistic-led critiques. First of all, autistic praxis in and of itself may be perceived as a challenge to neurotypically created societal structures. Bhaskar (2015) highlights that social agents’ praxis consists both of conscious production and, typically unconscious, reproduction of the structures that make up society. It is this unconscious reproduction of structures that autistic people do not typically partake in, to some degree. This is especially the case when one looks into the microsocial processes (Scheff, 2007) in autistic people’s lives, in other words the way that they navigate their day-to-day life. It may be less so the case when we examine macrosocial processes, how autistic people perceive larger social structures (Boatca, 2007), as autistic people are capable of having racist, sexist, homophobic, transphobic and ableist attitudes. Autistic people can, therefore, be just as unreflective about the role these larger social structures play in their lives and society more broadly as anybody else, particularly if they are not impacted by them directly.

Autistic people’s, often unconscious, resistance to the reproduction of social structures is, in my mind, both a core tenet of the autistic disposition and difficult to conceptualise. I believe that this autistic resistance to structures, which may be curbed throughout one’s lifetime both intentionally and unintentionally, is key to understanding the autistic disposition as it is manifested in the social world. Take capitalist economic structures for example. These typically dictate that most people must spend a third of their day doing some form of monetizable labour in some sort of workplace, to earn enough money to cover their basic necessities. Autistic people, many of whom are unable to sustain meaningful employment under the current neurotypical and capitalist regime, therefore, challenge this structure simply by existing (see also Milton, 2018; Yergeau, 2018), whether they want to or not, and have to live with the consequences of this for their entire lives, much like disabled people in general do (Oliver, 2004).

That is not to say, however, that autistic people do not reproduce some microsocial structures, in varying degrees; both in the case of masking/camouflaging as a survival mechanism adopted by autistic people and in the case of neurotypically-led behavioural interventions on autistic people, the very thing that is targetted is how to make autistic dispositions more compatible with the neurotypical world. As there is no such thing as an autistic society, there is no such thing as a mechanism that regulates autistic (or indeed neurotypical) relations that has been autistically created. Accounting for the second element of the agent-structure relation as well, the ever-present condition of (a neurotypically led/created) society is, therefore, crucial in understanding and conceptualising autism. We can, however, see a demonstration of autistic agency in the creation of autistic-led organisations and events, which show that autistic communities may create social norms that are liberating for autistic people that are participating in them (Sinclair, 2010) and challenge the way dominant neurotypical structures assert how spaces need to operate.

Understanding agent-structure relations is also crucial to conceptualise autistic emancipation. It is because autistic people are independent agents that they are able to enact their own emancipation and it is important to recognise them as such to be able to notice the multiplicity of ways in which autistic dispositions rebel against neurotypical structures. It is also important to recognise that autistic people inevitably change the structures they inhabit in a unique way because they are autistic and despite any neurotypical attempts to kerb their tendency to do that. If their autistic disposition were not what it is, the neurotypical world would not try to manage and control it. Existing as an autistic person, therefore, is almost a forceful demonstration in agency. As Bhaskar points out, social forms may change irrespective of the agent’s desire to change them in any particular way, yet it is important to recognise that social agents may also attempt to deliberately change the structures; there is a reason that so many autistic people become activists. It is also important to recognise the extensive pervasiveness of neurotypical societal structures. Autistic people may not even be able to perceive themselves outside of these ever-present and pervasive structures. For many autistic people, they even define how they perceive their own autistic disposition, making it impossible for them to conceptualise themselves away from the neurotypical gaze. Having a careful examination of the relationship between agency and structure, therefore, is key in understanding the various ways in which autistic sociality manifests itself.

### Critique, Explanation, Emancipation, and Autistic Participation in Autism Research

In the final part of this paper, I will present how the concepts of Critical Naturalism, namely explanation and emancipation, can help develop autism research that is based on and explanatory of, autism itself and how an ethical naturalist approach on autism research can help develop research that is simultaneously grounded in facts and ethically informed. Price (2019) presents the development of Bhaskar’s theory of explanation and emancipation in six levels. The first level is to identify that some belief we hold about an intransitive object is false; for example, the belief that autistic people do not possess theory of mind. The second level consists of applying the process from level one, instrumental rationality, in a particular context, such as a system of domination. For example, autistic people are perceived to lack theory of mind by neurotypical people, and thus the “theory of mind” approach is neurotypically created. At this level, it is also highlighted that there may be more than one problematic belief taking place. In the case of autism, factors like neurotypical assumptions about communication and capitalist structures that focus on monetary perceptions of efficiency and productivity also contribute to what is expected by autistic people in the first place, and therefore how their actions are judged as well. Level three consists of a negative evaluation of the false belief that accounts for the mismatch of the belief with the reality of what it is about. For example, stating that the belief that autistic people lack theory of mind is harmful and dehumanising, and also that in reality the difficulty autistic people have in empathising with non-autistic people is the same as the difficulty that non-autistic people have in empathising with autistic people.

Level four consists of positively evaluating actions that aim to disconnect the false belief from the object, actions that aim to challenge this pre-existing false consciousness. Continuing with the previous example, this would be designing research and practice that takes the double empathy problem into account and adopting an autism ethos that is informed by it. What this stage highlights about the uniqueness of theory for the social world is that the criticism of the belief will rub onto its cause, (Collier, 1994) which in this case would be a certain type of autism research and practice that it seeks to challenge. It is also worth noting that it is in this stage that the process will be faced with significant resistance; as Collier (1994) points out, certain institutions and false beliefs may be in a functional relation, as beliefs of false consciousness may serve to sustain such institutions in the first place. For example, research and funding that has been dedicated into further studying and exploring the lack of theory of mind in autistic people is directly challenged by this process and, should this premise be accepted, such research will have to significantly transform (and, in some cases, even be abandoned altogether). This is a significant challenge that will undoubtedly be met with resistance; however, if scientists are dedicated in pursuing the truth, as they ought to be, then this is a challenge they have to rise up to and adjust their practice accordingly.

Level five consists of a concrete ethical judgement of level four, which is specific to the geohistorical context that the theory was created in. Abstract universalism is, therefore, avoided and even the most powerful explanatory theory becomes a non-deterministic one (Buch-Hansen, 2005). In this way, the critical realist ontology demands a readjusting both in ethics and in epistemology. The stratified nature of reality helps us understand how a theory may be concrete at the level of the real, that of mechanisms, but not at the level of the empirical, that of experiences (Price, 2019). For example, simply because autistic people may be able to better empathise with each other, does not mean that they always do; A good example of that in the case of autism is racial, cultural, or ethnic differences; white autistic people may not always be able to empathise with autistic people of colour, and autistic people from different cultural backgrounds will have cultural barriers in the way of empathising with each other. It is important, therefore, to account for those differences when talking about the double empathy problem; this does not weaken the theory itself since it already recognises that these misunderstandings are, at least partly, cultural in the first place. Rather, it highlights the openness of the system it is applied to, the social world, in which no theory can be universal and deterministic. This is why self-reflexivity is always required as well; as Bhaskar highlights, critique is part of the process it describes because the very description it produces is subject to the same lack of reflexivity it identifies (Archer, 2010). Therefore, critical explanatory theory without self-reflection is just as moot as the theory it criticises.

The final level, level five, is the level in which the action occurs. Practical application of theories, therefore, and theory that informs practice, is how explanatory theories lead to emancipation under critical naturalism. It is by producing explanations that criticise social institutions that we begin the work of their subversion (Collier, 1994) and it is only when the subversion takes place that the process is complete. In this way, Critical Realism sets the roadmap for institutional change and sets a number of guidelines for evaluating the process.

The argument made here is that first of all, autism research ought to try to identify the truth about autism. In the earlier parts of the article, I have argued, I hope convincingly, that this may not happen without both significant autistic involvement in autism research and interdisciplinary approaches. The way autism research operates within current structures, however, may stand in the way of that, as not only do they not facilitate the two processes, but they also do not recognise their importance (Kapp et al., 2013; Milton and Bracher, 2013; Chown et al., 2017; Fletcher-Watson et al., 2019). It will need, therefore, to undergo significant transformation to meet this challenge and effectively produce research that investigates the truth about autism. Additionally, autistic emancipation is intrinsically tied to the recognition of autistic contributions, to autism knowledge that autistic people resonate with, and to the creation of policy and practice that is informed by such knowledge. This is what will transform the structures currently in place, within autism research, education, employment, social policy etc.

It is important to recognise that no research, regardless of its discipline, is completely asocial as all research is bound by the structure of the social world that encompasses it (Sayer, 1997). Commitment to social transformation, therefore, is everyone’s responsibility. Consequently, there needs to be recognition of how the current structures prevent autistic knowledge creation that is impactful. Furthermore, efforts toward autistic emancipation will always fight against larger systems that are fundamentally exclusive, such as capitalism. Given that impactful theory needs to first and foremost be practical, the argument here is not that no progress can be made unless these structures are first overthrown; rather, the argument I am making is that every autism scientist, irrespective of the field they work in, needs to have a basic understanding of how autism operates within neurotypical structures to be able to understand autism in the first place, and thus to be able to form meaningful research questions. Moreover, every autism scientist is responsible for the inclusion of autistic participation in their research if they intend for their research to be as close to the intransitive reality of autism as possible, and thus needs to be aware of the barriers that may prevent autistic people from making meaningful contribution to this process. For every autism research project there should be a concrete argument about how it aids autistic emancipation, and consequently autistic wellbeing, instead of reproducing structures whose knowledge production is inaccurate at least and harmful at most. Finally, every funding decision in autism research needs to justify how the research funded is beneficial, rather than harmful, to autistic people.

## Conclusion

In this article, I have attempted to summarise the main points of the philosophy of Critical Realism and demonstrate how it can be useful in critically assessing autism research, how it supports autistic participation in autism research and autistic theorising. I have attempted to show why a clear and consistent philosophy of autism, autistic participation in autism research as well as interdisciplinary approaches to knowledge production are crucial in the process of creating impactful autism research. To achieve that, however, the current structures around research and practice will need to be significantly transformed, and some even abandoned altogether. This will be a process that may be met with some resistance however, it is a necessary step forward to address the impasse that autism research finds itself into, and to shape autism research in a way that serves the interests of those that it is about, primarily autistic people and secondly those who live and work with them, parents, caregivers, professionals etc.

A variety of social barriers will have to be overcome for this to be achieved. Several academic disciplines may be inaccessible to autistic people for reasons that are beyond the particular institution’s immediate control, such as educational barriers that have prevented them to get the qualifications necessary to become researchers in the first place. There will be other barriers, however, that may be more easily addressed, such as providing an accessible workplace and creating space in the conversation for the autistic voice. Either way a collective, interdisciplinary commitment to autistic emancipation is the way forward and providing philosophically sound research is both a prerequisite and an outcome of it. Finally, although autistic emancipation should be a commitment for everyone producing autism research, even if that goal may sound too vague and political to some, ontologically and epistemologically sound research achieves just that; and Critical Realism is the vehicle to help achieve it. It is in every researcher’s best interest, therefore, to ensure that their research is ontologically resonant, epistemologically consistent, and ethically sound. This paper attempted to be the one of the first (see also Botha, 2021b), attempts in this endeavour.

## Data Availability Statement

The original contributions presented in the study are included in the article/supplementary material, further inquiries can be directed to the corresponding author.

## Author Contributions

The author confirms being the sole contributor of this work and has approved it for publication.

## Conflict of Interest

The author declares that the research was conducted in the absence of any commercial or financial relationships that could be construed as a potential conflict of interest. The reviewer MB declared a past co-authorship with the author MK.

## Publisher’s Note

All claims expressed in this article are solely those of the authors and do not necessarily represent those of their affiliated organizations, or those of the publisher, the editors and the reviewers. Any product that may be evaluated in this article, or claim that may be made by its manufacturer, is not guaranteed or endorsed by the publisher.

## Footnotes

1 Person first language is deliberately used here, to reflect the type of language often used by the neurotypical parents and professionals that engage in those types of arguments.
